# Efficacy of Emotion Regulation for Patients Suffering from Chronic Obstructive Pulmonary Disease

**Published:** 2017-01

**Authors:** Hongling WANG, Zhenhua WEI, Xue LI, Yongjie LI

**Affiliations:** 1. Dept. of Nursing, Linzi District People’s Hospital, Zibo Shandong Province, Zibo, PR China; 2. Dept. of Ophthalmology, Linzi District People’s Hospital, Zibo Shandong Province, Zibo, PR China; 3. Dept. of Outpatient, Central Hospital of Zibo, Shandong Province, Zibo, PR China

**Keywords:** Self-management program, Chronic obstructive pulmonary disease (COPD), Efficacy of emotion regulation

## Abstract

**Background:**

To study the influence values of self-management program intervention on efficacy of emotion regulation for patients suffering from chronic obstructive pulmonary disease (COPD).

**Methods:**

Eighty-six diagnosed chronic COPD patients in stable phase in Linzi District People’s Hospital, Zibo Shandong Province, PR China from June 2014 to June 2015 were selected in succession. They were divided into control group and observation group randomly with 43 cases in each group. In control group, conventional out-of-hospital continued nursing mode was used while strengthened self-management program guidance was used in observation group (including seven modules that included disease knowledge, breathing exercises, emotion management, home oxygen therapy, medicine intake technique, healthy life behaviors, and action plans in deterioration stage) to compare their differences of results.

**Results:**

For follow-up visits of 6 months, self-management behaviors of patients in two groups had increased, including physical fitness training, cognitive symptom management practice and medical care scores, and the increase range in observation group was more obvious and differences were of statistical significance (*P* < 0.05); self-efficacy of emotion regulation in two groups is increasing, including positive affect, despondency/distress, anger/irritation and total scores, furthermore, the increase range in observation group is more obvious and differences are of statistical significance (*P* < 0.05).

**Conclusion:**

Self-management program intervention can improve self-management behaviors of COPD patients and it is significant in terms of improving efficacy of emotion regulation and prognosis.

## Introduction

Chronic obstructive pulmonary disease (COPD) is a common respiratory disease clinically with high disability rate and fatality rate. COPD causes severe social psychosocial harm to patients because it cannot be treated over years (1). Self-efficacy of emotion regulation is an important index that evaluates confidence level and social competence of regulating individual emotion (2), and COPD patients need to well control the acute exacerbation process of the disease, meanwhile, keeping good emotion regulation ability and being positive and confident are very important (3).

Chronic disease self-management program has achieved successful application in education management of disease such as stroke, asthma, chronic bronchitis, and hypertension (4, 5), but there are few reports in terms of self-management intervention studies of COPD.

This study investigated self-management program intervention of COPD patients in order to provide scientific evidence for COPD patients in terms of health care and treatment. Please see reports as follows.

## Materials and Methods

### Object information

Eighty-six diagnosed COPD patients in stable phase in our hospital from June 2014 to June 2015 were selected in succession and the following cases were excluded from the study: uncontrolled in acute phase, lung cancer, lung abscess, severe pneumonia, respiratory failure, combined heart, brain, liver, kidney and other organs dysfunction, stable COPD symptoms less than 1 week, patients who could not take medicine and reexamine following the doctor’s advice and who cannot finish questionnaires.

This study has obtained the approval of the Ethics Committee from this hospital and informed consent of patients and their families.

Patients were divided into control group and observation group with each group 43 cases according to odd–even condition of last digital of admission number. For control group, there were 26 males and 17 females; their ages were between 44 and 78 years old and average was 62.6 ± 13.5 years old; their course of disease was 1∼8 year(s) and average was 3.2 ± 1.3 years; their education time was 13∼24 years and average was 18.6 ± 4.4 years. For observation group, there were 24 males and 19 females; their ages were between 45 and 76 years old and average was 61.8 ± 14.5 years old; their course of disease was 1.5∼10 years and the average was 3.5 ± 1.4 years; their education time was 12∼25 years and average was 17.6 ± 4.8 years. Baseline documents of two groups were comparable.

### Study method

Conventional out-of-hospital continued nursing mode was applied in control group, including establishing individual profile, regular follow-up phone call; guiding and supervising regular medicine intake outside the hospital such as Bronchodilators inhaled through mouth, glucocorticoid drugs, antibiotics and medicine relieving cough and asthma; guiding healthy lifestyle such as proper exercise, strengthening physique, preventing cold, quitting smoking and not actively or passively having secondhand smoke; long-term home oxygen therapy or community-based rehabilitation therapy could be applied for patients who had such conditions, evaluating symptoms and degree of COPD regularly and offering preventive suggestions and correct solutions for acute onset.

Strengthened self-management program guidance was used in observation group (including seven modules and they were disease knowledge, breathing exercises, emotion management, home oxygen therapy, medicine intake technique, healthy life behaviors, and action plans in deterioration stage). First, St George’s Respiratory Questionnaire testing and COPD health knowledge questionnaire were used. Medicine treatment shall be according to investigation conclusion and patients’ diseases severity. Meanwhile, personalized self-management program intervention scheme and related course guidelines would be prepared by respiration medicine experts and specialized nurses. Specialized nurses and nursing staff from community health service center shall establish long-term communication mechanism with patients and visit them regularly, guiding their self-management and pointing out and correcting mistakes in time. They can use books and videos to demonstrate and explain to patients and let patients practice repeatedly till they have acquired above knowledge and skills. Follow-up visit shall be once a week and 1.5 h each time, and then it shall be changed to follow-up phone calls 8 weeks later and the course of intervention is 6 months.

### Observation indexes

To evaluate the differences of self-management behaviors before intervention and last 6 months with scale scores of self-efficacy of emotion regulation. For self-management behavior, evaluation was carried out in accordance with self-management behavior questionnaire and scoring standards and Chronic Self-management Study Measurement Table (6); questionnaire included three quantitative indexes and they were exercise training, cognitive symptom management practice, and medical care with 15 items in total; five-grading system (0–4 points) was used for exercise training measurement table and six-grading system (0–5 points) was used for the other two. Total score of measurement table was according to the average score of items, the higher the score was, the more effective the self-management behavior was. Self-efficacy of emotion regulation evaluation used SRESE measurement table (Caprara 2008 Edition) (7), including three dimensions and they were perceived self-efficacy in expressing positive affect (POS), perceived self-efficacy in managing despondency/distress (DES), and perceived self-efficacy in managing anger/irritation (ANG). There were 12 items in questionnaire and 5-point scoring system (1–5 points) was used. Total score was the sum of scores in all dimensions. The higher the score was, the better the self-efficacy of emotion regulation was.

### Statistical method

SPSS 19.0 software (SPSS Inc., Chicago, IL, USA) was used in data analysis; measurement data are presented as the mean ± standard deviation and comparison between groups was tested by an independent t-sample and comparison within group is tested by matched *t*; enumeration data are presented as the number of cases or the percentage (%) and comparison between groups was tested by χ^2^. *P* < 0.05 indicates that differences are of statistical significance.

## Results

Comparisons of self-management behaviors before intervention were in terms of physical fitness training, cognitive symptom management practice, and medical care scores and there were no statistical significances regarding their differences (*P* > 0.05); scores of all the above-mentioned indexes of patients in two groups in follow-up visit after 6 months had increased; furthermore, the increase range in observation group was more significant and the differences were of statistical significance (*P* < 0.05) (Table 1).

**Table 1:** Self-management behavior scores in two groups

**Group**		**Physical fitness training**	**Cognitive symptom management practice**	**Medical care**
Observation group	Before intervention	7.2 ± 4.7	1.4 ± 0.8	0.9 ± 0.3
	6 months later	19.0 ± 5.3	2.9 ± 0.5	2.3 ± 0.6
Control group	Before intervention	7.3 ± 5.0	1.4 ± 0.5	1.0 ± 0.2
	6 months later	10.3 ± 7.5	1.7 ± 0.6	1.9 ± 0.7

Comparisons of self-efficacy of emotion regulation before intervention were in terms of POS, DES, ANG, and total scores and differences were of no statistical significance (*P* > 0.05); scores of all the above-mentioned indexes of patients in two groups in follow-up visit after 6 months had increased; furthermore, the increase range in observation group was more significant and the differences were of statistical significance (*P* < 0.05) (Table 2).

**Table 2:** Self-efficacy of emotion regulation in two groups

**Group**		**POS**	**DES**	**ANG**	**Total scores**
Intervention group	Before intervention	9.0 ± 2.0	7.8 ± 3.5	8.2 ± 2.5	25.2 ± 3.0
	6 months later	17.0 ± 3.8	15.8 ± 2.1	15.0 ± 4.1	45.5 ± 5.5
Control group	Before intervention	9.3 ± 1.6	7.7 ± 4.0	8.3 ± 3.4	25.1 ± 4.8
	6 months later	11.8 ± 4.2	10.8 ± 2.9	10.3 ± 4.9	33.2 ± 5.6

## Discussion

Efficacy of emotion regulation involves in many aspects of healthy life and has significant influence on mental health of patients, including positive and supported attitude towards disease treatment and on their tolerance to harmful trends such as anxiety and depression (8). Therefore, efficacy of emotion regulation is significant in COPD treatment.

Self-management behavior refers to behaviors that prevent diseases from development, monitor signs of disease and improve life quality, long-term good self-management behaviors are good for developing healthy lifestyles, increasing adaptive behaviors, relieving discomfort symptoms and even improving body’s immune function (9). Self-management program intervention can help COPD patients learn disease management knowledge and skills as well as improving patients’ positive attitude toward disease, which has been widely approved by medical circles home and abroad (10). COPD is hard to treat, so patients are easily getting anxious, depressed, negative and disappointed, which are reasons why COPD patients cannot positively face their diseases and they “surrender” to it. This will surely prevent them from developing effective disease management behaviors and furthermore, influence self-efficacy of emotion regulation and improvement of life quality (11). It has been revealed in the study (12) that for follow-up visits of COPD patients discharged from the hospital for half a year, the incidence rates of acute exacerbation have increased, which are significantly related to poor self-efficacy of emotion regulation. In this study, efficacy scores of emotion regulation for patients in two groups are low, indicating that COPD patients have significant problems in regulating depression/pain/anger emotions. With great improvement of self-management behaviors in observation group, its self-efficacy of emotion regulation has also increased significantly and all indexes are getting better when patients discharge from hospital compared with control group. It indicates that self-management program intervention can help patients face disease positively, motivate their potentials, and improve disease management ability in order to improve their life quality and self-efficacy of emotion regulation.

In addition, organizing chronic disease self-management in form of groups is good for arousing the enthusiasm and interactivity of patients, which is able to improve significantly clinical prognosis (13). Due to the particularity of COPD disease, patients suffer for a long-time torment and most of them have severe asthma after activity, but it is hard to gather them and educate. Therefore, in this study, individualized education and face-to-face guidance are combined in observation group with family visit, which is easy for patients to accept, the guidance is more complete and thorough, and it helps patients solve psychosocial problems better (14).

## Conclusion

Self-management program intervention is the most potential treatment resource in recovery indexes of COPD patients, which guides patients to carry out rehabilitation exercise and disease management in stable phase actively and effectively according to schemes and is good for improvement of self-efficacy of emotion regulation.

## Ethical considerations

Ethical issues (Including plagiarism, informed consent, misconduct, data fabrication and/or falsification, double publication and/or submission, redundancy, etc.) have been completely observed by the authors.
